# Association between added sugars and kidney stones in U.S. adults: data from National Health and Nutrition Examination Survey 2007–2018

**DOI:** 10.3389/fnut.2023.1226082

**Published:** 2023-08-04

**Authors:** Shan Yin, Zhenzhen Yang, Pingyu Zhu, Zhongbo Du, Xiaodong Yu, Tielong Tang, Yan Borné

**Affiliations:** ^1^Department of Urology, Affiliated Hospital of North Sichuan Medical College, Nanchong, China; ^2^Department of Clinical Laboratory, Nanchong Central Hospital, Nanchong, China; ^3^Nutritional Epidemiology, Department of Clinical Sciences Malmö, Lund University, Malmö, Sweden

**Keywords:** adult, association, dietary sugars, nephrolithiasis, nutrition surveys

## Abstract

**Purpose:**

Added sugar is associated with a variety of adverse health outcomes, but its association with kidney stones is unclear. This study was to determine whether added sugar is associated with kidney stones.

**Materials and methods:**

This nationally representative study used National Health and Nutrition Examination Survey (NHANES) datasets from 2007 to 2018 for analysis. People aged ≥20 years who reported a history of kidney stones and provided dietary recall data on added sugars were included. Weighted proportions, multivariable logistic regression analysis and stratified logistic regression were used to evaluate the associations between added sugars and kidney stones by adjusting potential confounders.

**Results:**

Totally 28,303 adults were included, with weighted mean age [95% confidence interval (CI)] of 48.03 (47.56, 48.51) years, 47.74% (47.09, 48.40%) males and 52.26% (51.60, 52.91%) females. The overall mean (95% CI) energy intake from added sugars was 272.10 (266.59, 277.60) kilocalories. In the fully-adjusted multivariable model, the percentage of energy intake from added sugars was positively correlated with kidney stones. Compared to the first quartile of added sugar energy intake percentage, the population in the fourth quartile had a higher prevalence of kidney stones (OR = 1.39; 95% CI 1.17 to 1.65). Compared with the less than 5% calories from added sugar population, the more than or equal to 25% calories from added sugar had a higher kidney stone prevalence (OR = 1.88; 95% CI 1.52 to 2.32).

**Conclusion:**

A higher percentage of energy intake from added sugars is significantly associated with a higher prevalence of kidney stones. This study provides cross-sectional evidence for the relationship between added sugars and health outcomes.

## Introduction

Kidney stones are a common disease worldwide, affecting about 1 in 10 people in the United States (1). The occurrence of kidney stones is increasing, which puts a huge strain on the healthcare system (2). Kidney stones have a high recurrence rate, with about 50% of people experiencing a second episode within 10 years (3). Despite this, no clear mechanism of kidney stone etiology has been defined yet. Therefore, further research is needed to identify potential intervention targets and explore the underlying mechanism and potential risk factors. According to recent epidemiologic research, diet (4) and lifestyle factors (2), may play an important role in kidney stones development or prevention.

Sugars or caloric sweeteners added to foods or beverages during processing or preparation to add flavor or extend shelf life are considered added sugars. Sugar-sweetened beverages (SSBs) such as soda and energy and sports drinks were the largest food group sources of added sugars, accounting for 34.4% in the American diet (5). In previous studies, consumption of added sugars from SSBs was associated with a higher risk of metabolic disorder such as obesity (6, 7), diabetes (8, 9), and cardiovascular disease (CVD) (9, 10). These diseases are closely related to kidney stones (11–14). Nevertheless, there is limited evidence revealing the relationship between added sugars and kidney stones.

Considering the negative health effects of added sugar, various agencies recommend limiting its intake and have set different upper limits. The American Heart Association (AHA) recommended that the daily average intake from added sugars no more than 150 kcal for adult male and no more than 100 kcal for female (15). Recommendations of the Institute of Medicine (IOM) allow up to 25% of calories to be consumed from added sugars (16). According to 2020 Dietary Guidelines for Americans (DGA) (17) and World Health Organization (WHO) (18, 19), 10 percent of calories from added sugar per day is their recommended upper limit. WHO suggests a conditional recommendation to below 5% of total energy intake (19).

Added sugars are associated with multiple adverse outcomes, and there is controversy regarding the appropriate upper limit for added sugar consumption. In addition, the relationship between added sugars and kidney stones is still unclear, and there is a lack of nationally representative sample studies. Moreover, previous studies have mainly focused on the relationship between beverages and kidney stones (20, 21), and there is a lack of evidence on the relationship between total added sugar intake and kidney stones. Therefore, we examined the association between added sugars and kidney stones using the large population data from the National Health and Nutrition Examination Survey (NHANES). We hypothesize that more calorie intake from added sugar may be associated with a higher prevalence of kidney stones.

## Materials and methods

### Study population

NHANES is a program of studies designed to evaluate participants’ health and nutritional status in America. It combines interviews and physical examinations. In 1999, the survey became a continuous program that has a changing focus on a variety of health and nutrition measurements to meet emerging needs, with every 2 years representing one cycle. The survey examines a nationally representative sample of about 5,000 persons each year. We used six continuous cycles of NHANES data from year 2007 to 2018, which contain data for both kidney stones and added sugars.

This cross-sectional study included participants aged 20 years or older (*n* = 34,770). Then pregnant participants (*n* = 372) and dietary recall not reliable or incomplete information on kidney stones or first day added sugars intake (*n* = 4,030) were excluded. Moreover, female participants with energy intake <600 or >3,500 kcal or male participants with energy intake <800 or >4,200 kcal (*n* = 2065) were excluded. Finally, 28,303 eligible people were included for further analyses. The detailed inclusion and exclusion criteria were shown in Supplementary Figure S1.

All NHANES study protocols were approved by the National Center for Health Statistics (NCHS) research ethics review board and by all participants. This cross-sectional study followed the Strengthening the Reporting of Observational Studies in Epidemiology (STROBE) reporting guidelines (22).

### Outcome and exposure assessment

The main outcome was if the participant ever had kidney stones. In this study, kidney stone was identified through the participants’ self-reports. In NHANES questionnaire sections, participants who replied “yes” to “Have you/Has sample person (SP) ever had kidney stones?” were considered to have a history of kidney stones.

The major exposure factor was added sugars, which are defined as sugars that are added to foods as an ingredient during preparation, processing, or at the table, and do not include naturally occurring sugars such as lactose present in milk and fructose present in fruits (23). In NHANES, added sugars include brown sugar, cane syrup, corn syrups, corn syrup solids, dextrose, fructose, fruit syrups, honey, maple syrup, molasses, pancake syrups, raw sugar, sorghum syrups, and white sugar. We used 24 h dietary recall to estimate intake of added sugar. All NHANES participants are eligible for two 24 h dietary recall interviews. The first dietary recall interview is collected in-person in the Mobile Examination Center (MEC) and the second interview is collected by telephone 3 to 10 days later. Dietary data (including total energy and added sugars) was extracted from the total nutrient intakes on the first day (DR1TOT), the second day (DR2TOT) and United States Department of Agriculture (USDA) MyPyramid Equivalents Database/Food Patterns Equivalents Database (MPED/FPED) files. The teaspoon equivalent (tsp) values of added sugars in the FPED were transformed into grams (4.2 g/tsp) and kilocalories (3.87 kcal/g). Given that an individual’s dietary consumption is closely related to body size, metabolic efficiency, and physical activity, we used the added sugar energy percentage (daily energy from added sugars divided by total daily energy) for all analyses. We classified the percentage of added sugar energy intake by two methods: the first was divided into quartiles; the second was divided according to the cutoff value recommended by different institutions, divided percentage into <5, 5–10, 10–25, and ≥25. In addition, according to the website of NAHENS, since the response rate for dietary recall on day 1 was relatively higher compared to day 2, our main analysis was to use dietary data from day 1. We also performed a sensitivity analysis using dietary data from day 2 or the average intake over the 2 days.

### Assessment of covariates

We adjusted demographic covariates: age (categorized as 20–34, 35–49, 50–64, and ≥65 years), gender (male, female), race (Mexican American, other Hispanic, non-Hispanic white, non-Hispanic black, other races), marital status (married, widowed, divorced, separated, never married, and living with partner), education (less than 9th grade, 9–11th grade, high school graduate, some college, and college graduate or above), poverty income ratio (PIR, categorized as ≤1.3, >1.3 & ≤3.5, >3.5); anthropometric measure such as body mass index (BMI, a measure of body fat based on an individual’s weight in kilograms divided by the square of their height in meters, categorized as <25, 25–30, and ≥30 kg/m^2^); personal life styles such as smoking (never: smoked less than 100 cigarettes in life; former: smoked more than 100 cigarettes in life and smoke not at all now; current: smoked moth than 100 cigarettes in life and smoke some days or every day currently), alcohol (drinker: ≥12 alcohol drinks per year; non-drinker: <12 alcohol drinks per year), physical activity (physical activity intensity was measured by multiples of the metabolic equivalent of task (MET), 1 week total MET-minutes of one participant was sum of work activity (days vigorous work × minutes vigorous-intensity work × 8, days moderate work × minutes moderate-intensity work × 4), recreational activities (days vigorous recreational activities × minutes vigorous recreational activities × 8, days moderate recreational activities × minutes moderate recreational activities × 4), and walk or bicycle (number of days walk or bicycle × minutes of walk or bicycle for transportation × 4), then categorized as <500 or ≥500 MET-minutes/week); some dietary assessment such as daily total energy intake and healthy eating index 2015 score (HEI-2015). HEI-2015 is a dietary index used to evaluate the quality of one’s diet. It consists of 13 components: nine adequacy components and four moderation components, with a maximum score of 100 points. Each component has a specific maximum score between 5 and 10 points. The specific algorithm for calculating HEI-2015 scores has been elaborated in previous study (4); and some self-reported chronic conditions (all classified as yes/no): gout, stroke, diabetes (diagnosed by any of the following: told by a doctor or health professional; glycohemoglobin ≥6.5%; fasting blood sugar ≥7.0 mmol/L; random blood sugar ≥11.1 mmol/L; two-hour glucose (oral glucose tolerance test, OGTT) ≥11.1 mmol/L; use of antidiabetic medications. Impaired glucose tolerance and impaired fasting glycaemia and those who self-reported “borderline” were considered to not have diabetes), hypertension (diagnosed by any of the following: told by a doctor or health professional; use of antihypertensive drugs; systolic blood pressure ≥140 mmHg or/and diastolic pressure ≥90 mmHg), cardiovascular disease (CVD, at least one of these heart diseases: coronary heart disease, angina, congestive heart failure) and cancer; In addition, we also included year cycle as a covariate. Dummy variables were used to indicate missing covariate values for variables with missing values greater than 2%.

### Statistical analysis

We used complex sampling weights (MEC exam weight) recommended by Centers for Disease Control and Prevention (CDC). And we combined the sample weights of 6 continuous cycles according to the recommended method on the NHANES website.^1^ In the baseline characteristics table, continuous variables were expressed as survey-weighted mean [95% confidence interval (CI)], and categorical variables as survey-weighted percentage (95% CI).

To explore the association between added sugars and kidney stones, we used three logistic regression models with or without adjustment of covariates. Model 1 was unadjusted. Model 2 was adjusted for age, gender and race. Model 3 was adjusted for gender, age, race, PIR, BMI, education, marital status, smoking, alcohol, physical activity, HEI-2015, energy, gout, stroke, diabetes, hypertension, CVD, cancer and year cycle. To better explore the association between added sugars and kidney stones, multivariable logistic regression was conducted to explore added sugars energy percentage as continuous and categorical variables (divided into quartiles and divided into four categories according to 5%, 10%, and 25% added sugars energy percentage). The trends were estimated by treating added sugars energy percentage categories as a continuous variable. Then to test whether there was a non-linear association between added sugars energy percentage and kidney stones, we performed spline smoothing with a generalized additive model (GAM) and a piecewise linear regression. In order to further analyze the relationship between added sugars and kidney stones, two sensitivity analyses were conducted. In the first analysis, data from the second day of dietary recall was used in a multivariable logistic regression analysis. In the second analysis, the mean dietary intake over the 2 days was used in a multivariable logistic regression analysis. Finally, we further used stratified logistic regression models according to all potential confounding factors at the baseline table.

All analyses were performed using R 4.2.0 (http://www.R-project.org; The R Foundation) and EmpowerStats (http://www.empowerstats.com, X&Y Solutions, Inc.). A two-tailed *p* < 0.05 was considered statistically significant.

## Results

### Population, outcome and exposure factors characteristics

Supplementary Figure S1 described the inclusion and exclusion criteria. A total of 28,303 American adults were included, with weighted mean age (95% CI) of 48.03 (47.56, 48.51) years, and 47.74% (47.09%, 48.40%) were males and 52.26% (51.60%, 52.91%) were females. Table 1 presented the baseline population characteristics according to the quartiles of added sugar energy percentage. The overall prevalence of kidney stones was 10.13% (95% CI, 9.64 to 10.65%). The overall mean (95% CI) daily energy was 2065.42 (2050.91, 2079.92) kcal, and energy from added sugar was 272.10 (266.59, 277.60) kcal. An increased intake of added sugar percentage corresponds to higher prevalence of kidney stones, lower HEI score and lower education level.

**Table 1:** Characteristics of participants by categories of added sugars energy percentage: NHANES 2007–2018.

	All mean (95% CI)	*Q*1 (0.00–5.00)	*Q*2 (6.00–10.00)	*Q*3 (11.00–17.00)	*Q*4 (18.00–97.00)
Age (years)	48.03 (47.56, 48.51)	49.14 (48.43, 49.86)	49.95 (49.26, 50.63)	48.20 (47.57, 48.83)	45.06 (44.44, 45.67)
*Age category (%)*					
20–34	26.46 (25.34, 27.62)	24.45 (22.75, 26.23)	22.63 (20.95, 24.40)	26.44 (24.94, 28.01)	31.93 (30.14, 33.78)
35–49	26.99 (26.03, 27.97)	25.43 (24.10, 26.81)	25.68 (24.05, 27.38)	26.97 (25.31, 28.69)	29.65 (28.30, 31.04)
50–64	27.21 (26.33, 28.10)	28.92 (27.41, 30.48)	29.42 (27.62, 31.30)	26.58 (24.98, 28.24)	24.21 (22.77, 25.72)
≥65	19.34 (18.47, 20.24)	21.21 (19.77, 22.71)	22.27 (20.75, 23.86)	20.01 (18.72, 21.37)	14.20 (13.08, 15.40)
PIR	3.03 (2.96, 3.09)	3.22 (3.14, 3.31)	3.24 (3.15, 3.33)	3.04 (2.96, 3.12)	2.63 (2.54, 2.72)
*PIR category (%)*					
≤1.3	19.62 (18.44, 20.86)	17.09 (15.78, 18.49)	16.57 (15.27, 17.95)	18.40 (16.92, 19.97)	26.04 (24.18, 27.99)
>1.3 and ≤3.5	33.10 (31.83, 34.40)	30.59 (28.63, 32.62)	30.74 (28.85, 32.70)	33.92 (32.19, 35.69)	36.74 (35.09, 38.43)
>3.5	40.07 (38.22, 41.96)	44.74 (42.35, 47.14)	45.55 (43.11, 48.01)	40.35 (38.08, 42.65)	30.43 (28.23, 32.73)
Missing	7.20 (6.60, 7.86)	7.58 (6.56, 8.74)	7.14 (6.23, 8.18)	7.34 (6.46, 8.33)	6.78 (6.00, 7.66)
BMI (kg/m^2^)	29.10 (28.92, 29.28)	29.08 (28.77, 29.39)	28.89 (28.62, 29.16)	29.16 (28.88, 29.44)	29.27 (29.05, 29.48)
*BMI category (%)*					
<25 kg/m^2^	29.38 (28.29, 30.50)	28.76 (26.93, 30.66)	29.55 (27.82, 31.35)	29.95 (28.24, 31.72)	29.19 (27.58, 30.86)
≥25, <30 kg/m^2^	32.87 (31.99, 33.77)	33.24 (31.53, 35.01)	34.65 (33.29, 36.04)	31.90 (30.28, 33.57)	31.84 (30.32, 33.39)
≥30 kg/m^2^	37.74 (36.59, 38.91)	38.00 (35.92, 40.12)	35.79 (34.05, 37.57)	38.15 (36.33, 40.00)	38.97 (37.49, 40.47)
Physical activity (MET-minutes/week)	4749.99 (4585.23, 4914.75)	4296.84 (4039.79, 4553.90)	4223.45 (4011.79, 4435.11)	4704.27 (4433.09, 4975.46)	5739.92 (5501.64, 5978.19)
*Physical activity (%)*					
<500 MET-minutes/week	12.02 (11.58, 12.48)	11.92 (10.90, 13.03)	11.95 (10.98, 13.00)	12.60 (11.58, 13.69)	11.59 (10.70, 12.55)
≥500 MET-minutes/week	65.52 (64.55, 66.48)	68.12 (66.43, 69.75)	65.62 (64.06, 67.16)	64.77 (63.03, 66.47)	63.93 (62.59, 65.25)
Missing	22.45 (21.54, 23.39)	19.96 (18.52, 21.49)	22.42 (20.90, 24.03)	22.63 (21.16, 24.18)	24.48 (23.29, 25.71)
HEI-2015	51.10 (50.67, 51.53)	55.55 (55.00, 56.09)	54.84 (54.22, 55.47)	50.71 (50.23, 51.19)	44.00 (43.59, 44.42)
Energy (kcal)	2065.42 (2050.91, 2079.92)	1929.66 (1901.98, 1957.35)	2065.65 (2040.12, 2091.18)	2129.46 (2107.49, 2151.44)	2118.76 (2096.81, 2140.71)
Added sugars (kcal)	272.10 (266.59, 277.60)	58.25 (56.79, 59.70)	165.14 (162.92, 167.36)	292.25 (288.92, 295.58)	542.11 (533.05, 551.17)
Total sugars (kcal)	416.66 (411.94, 421.38)	214.08 (208.66, 219.50)	327.09 (321.67, 332.51)	442.25 (436.66, 447.83)	654.48 (645.63, 663.32)
*Gender (%)*					
Female	52.26 (51.60, 52.91)	49.62 (47.84, 51.40)	52.84 (51.33, 54.35)	53.61 (52.25, 54.97)	52.62 (51.21, 54.02)
Male	47.74 (47.09, 48.40)	50.38 (48.60, 52.16)	47.16 (45.65, 48.67)	46.39 (45.03, 47.75)	47.38 (45.98, 48.79)
*Race (%)*					
Mexican American	8.27 (6.93, 9.84)	8.12 (6.70, 9.80)	7.90 (6.54, 9.53)	8.75 (7.26, 10.52)	8.26 (6.75, 10.05)
Other Hispanic	5.67 (4.82, 6.66)	5.40 (4.51, 6.46)	5.61 (4.74, 6.62)	6.21 (5.19, 7.42)	5.42 (4.52, 6.49)
Non-Hispanic white	67.55 (64.75, 70.24)	68.05 (65.26, 70.72)	69.47 (66.71, 72.09)	66.75 (63.71, 69.65)	66.10 (62.35, 69.67)
Non-Hispanic black	10.82 (9.50, 12.30)	8.00 (6.99, 9.14)	8.76 (7.66, 9.99)	11.12 (9.65, 12.79)	14.99 (12.89, 17.36)
Other races	7.68 (6.89, 8.56)	10.43 (9.09, 11.95)	8.27 (7.13, 9.57)	7.16 (6.26, 8.18)	5.23 (4.52, 6.05)
*Education (%)*					
Less than 9th grade	5.10 (4.58, 5.68)	6.00 (5.27, 6.84)	4.65 (4.03, 5.36)	4.93 (4.29, 5.65)	4.92 (4.30, 5.63)
9–11th grade	10.07 (9.26, 10.94)	8.70 (7.81, 9.68)	8.53 (7.67, 9.48)	9.08 (8.18, 10.07)	13.77 (12.37, 15.30)
High school graduate	23.02 (21.94, 24.13)	19.24 (17.69, 20.88)	20.59 (19.09, 22.18)	22.72 (21.25, 24.26)	28.97 (27.41, 30.58)
Some college	31.31 (30.27, 32.36)	28.85 (27.33, 30.41)	28.94 (27.18, 30.77)	32.73 (31.16, 34.35)	34.28 (32.64, 35.96)
College or above	30.51 (28.60, 32.48)	37.21 (34.72, 39.78)	37.29 (34.55, 40.11)	30.54 (28.45, 32.71)	18.06 (16.36, 19.89)
*Marital (%)*					
Married	55.64 (54.22, 57.05)	58.23 (56.08, 60.35)	59.92 (57.85, 61.96)	55.54 (53.49, 57.57)	49.34 (47.06, 51.62)
Widowed	5.90 (5.54, 6.27)	6.22 (5.56, 6.96)	6.56 (5.86, 7.34)	6.00 (5.40, 6.67)	4.86 (4.31, 5.46)
Divorced	10.20 (9.65, 10.78)	9.47 (8.52, 10.52)	9.35 (8.32, 10.49)	9.97 (8.98, 11.05)	11.90 (10.95, 12.93)
Separated	2.33 (2.09, 2.60)	2.01 (1.62, 2.48)	1.83 (1.46, 2.29)	2.28 (1.90, 2.74)	3.14 (2.71, 3.64)
Never married	18.01 (16.88, 19.20)	16.63 (14.99, 18.41)	16.12 (14.78, 17.55)	18.11 (16.69, 19.62)	20.94 (19.23, 22.75)
Living with partner	7.93 (7.40, 8.49)	7.43 (6.40, 8.61)	6.22 (5.42, 7.15)	8.10 (7.28, 9.01)	9.83 (8.98, 10.75)
*Alcohol (%)*					
Non-drinker	17.95 (16.82, 19.13)	16.08 (14.74, 17.51)	16.96 (15.57, 18.45)	18.90 (17.36, 20.55)	19.56 (18.05, 21.16)
Drinker	60.37 (58.77, 61.94)	61.65 (59.39, 63.86)	59.44 (57.13, 61.71)	60.08 (57.92, 62.21)	60.41 (58.13, 62.65)
Missing	21.69 (20.40, 23.04)	22.27 (20.46, 24.19)	23.60 (21.53, 25.79)	21.01 (19.23, 22.92)	20.03 (17.81, 22.45)
*Smoke (%)*					
Never	56.20 (55.04, 57.35)	57.02 (54.90, 59.12)	58.59 (56.53, 60.63)	59.00 (57.47, 60.51)	50.31 (48.41, 52.22)
Former	24.94 (24.00, 25.90)	28.13 (26.43, 29.89)	27.83 (26.12, 29.61)	24.04 (22.85, 25.27)	20.28 (18.81, 21.83)
Current	18.86 (17.96, 19.80)	14.85 (13.52, 16.27)	13.58 (12.32, 14.93)	16.96 (15.88, 18.10)	29.41 (27.82, 31.05)
*Gout (%)*					
No	95.87 (95.52, 96.20)	94.82 (93.86, 95.64)	95.76 (95.05, 96.37)	96.47 (95.90, 96.96)	96.30 (95.71, 96.81)
Yes	4.13 (3.80, 4.48)	5.18 (4.36, 6.14)	4.24 (3.63, 4.95)	3.53 (3.04, 4.10)	3.70 (3.19, 4.29)
*Cancer (%)*					
No	89.29 (88.76, 89.79)	89.09 (87.97, 90.12)	88.15 (87.03, 89.18)	89.34 (88.38, 90.23)	90.50 (89.64, 91.29)
Yes	10.71 (10.21, 11.24)	10.91 (9.88, 12.03)	11.85 (10.82, 12.97)	10.66 (9.77, 11.62)	9.50 (8.71, 10.36)
*Diabetes (%)*					
No	85.30 (84.63, 85.94)	80.60 (79.13, 82.00)	84.14 (82.81, 85.38)	86.74 (85.52, 87.87)	89.07 (88.25, 89.84)
Yes	14.70 (14.06, 15.37)	19.40 (18.00, 20.87)	15.86 (14.62, 17.19)	13.26 (12.13, 14.48)	10.93 (10.16, 11.75)
*Hypertension (%)*					
No	61.39 (60.27, 62.51)	58.97 (56.89, 61.03)	60.20 (58.31, 62.07)	62.63 (60.93, 64.30)	63.40 (61.86, 64.91)
Yes	38.61 (37.49, 39.73)	41.03 (38.97, 43.11)	39.80 (37.93, 41.69)	37.37 (35.70, 39.07)	36.60 (35.09, 38.14)
*Stroke (%)*					
No	96.90 (96.63, 97.16)	97.23 (96.77, 97.63)	96.55 (96.03, 97.00)	97.31 (96.89, 97.68)	96.55 (96.00, 97.03)
Yes	3.10 (2.84, 3.37)	2.77 (2.37, 3.23)	3.45 (3.00, 3.97)	2.69 (2.32, 3.11)	3.45 (2.97, 4.00)
*CVD (%)*					
No	91.02 (90.47, 91.55)	90.52 (89.50, 91.45)	90.11 (89.10, 91.03)	91.92 (91.22, 92.57)	91.42 (90.52, 92.25)
Yes	8.98 (8.45, 9.53)	9.48 (8.55, 10.50)	9.89 (8.97, 10.90)	8.08 (7.43, 8.78)	8.58 (7.75, 9.48)
*Kidney stones (%)*					
No	89.87 (89.35, 90.36)	90.37 (89.21, 91.42)	90.63 (89.65, 91.51)	90.26 (89.28, 91.17)	88.29 (87.22, 89.29)
Yes	10.13 (9.64, 10.65)	9.63 (8.58, 10.79)	9.37 (8.49, 10.35)	9.74 (8.83, 10.72)	11.71 (10.71, 12.78)

For continuous variables: survey-weighted mean (95% CI). For categorical variables: survey-weighted percentage (95% CI).

PIR, poverty income ratio; BMI, body mass index; CVD, cardiovascular disease.

### Multivariate regression analysis

Multivariate regression analysis showed that added sugar percentage was positively correlated with kidney stones. After adjustment for multiple confounders (OR = 1.02; 95% CI 1.01 to 1.02). Based on the quartiles of added sugar percentage, all three models showed a positive correlation between added sugar percentage and the prevalence of kidney stones. Compared with the first quartile of the added sugar percentage population, the fourth quartile had a higher kidney stone prevalence in fully adjusted model (OR = 1.39; 95% CI 1.17 to 1.65). *p* value for trend <0.001 (Table 2).

**Table 2:** Association of added sugars energy percentage with kidney stones.

Exposure	Model 1^a^	Model 2^b^	Model 3^c^
%kcal added sugars (continuous)	1.01 (1.01, 1.02)	1.02 (1.01, 1.02)	1.02 (1.01, 1.02)
*Quartile of %kcal added sugars*			
*Q*1 (0.00–5.00)	1.0 (Ref)	1.0 (Ref)	1.0 (Ref)
*Q*2 (6.00–10.00)	0.97 (0.81, 1.17)	0.96 (0.80, 1.16)	1.00 (0.82, 1.21)
*Q*3 (11.00–17.00)	1.01 (0.87, 1.17)	1.06 (0.92, 1.23)	1.11 (0.95, 1.29)
*Q*4 (18.00–97.00)	1.24 (1.08, 1.44)	1.430 (1.23, 1.67)	1.39 (1.17, 1.65)
*p*-value for trend	0.002	<0.001	<0.001
*Percentage of energy from added sugars*			
<5	1.0 (Ref)	1.0 (Ref)	1.0 (Ref)
5–10	1.05 (0.88, 1.25)	1.03 (0.86, 1.23)	1.11 (0.92, 1.33)
10–25	1.10 (0.95, 1.27)	1.17 (1.01, 1.36)	1.23 (1.06, 1.44)
>25	1.60 (1.33, 1.93)	1.93 (1.59, 2.35)	1.88 (1.52, 2.32)
*p*-value for trend	<0.001	<0.001	<0.001

PIR, poverty income ratio; BMI, body mass index; CVD, cardiovascular disease.

a Non-adjusted model: adjusted for none.

b Minimally adjusted model: adjusted for gender, age, race.

c Fully adjusted model: adjusted for gender, age, race, PIR, BMI, education, marital status, smoking, alcohol, energy, HEI-2015, physical activity, gout, diabetes, hypertension, stroke, CVD, cancer, and year cycle.

Compared with the less than 5% calories from added sugar population, the more than or equal to 25% calories from added sugar had a higher kidney stone prevalence in model 1 (OR = 1.60; 95% CI 1.33 to 1.93), model 2 (OR = 1.93; 95% CI 1.59 to 2.35) and model 3 (OR = 1.88; 95% CI 1.52 to 2.32) (*p* for trend <0.001) (Table 2).

### Spline smoothing and piece-wise regression

**Figure 1:**
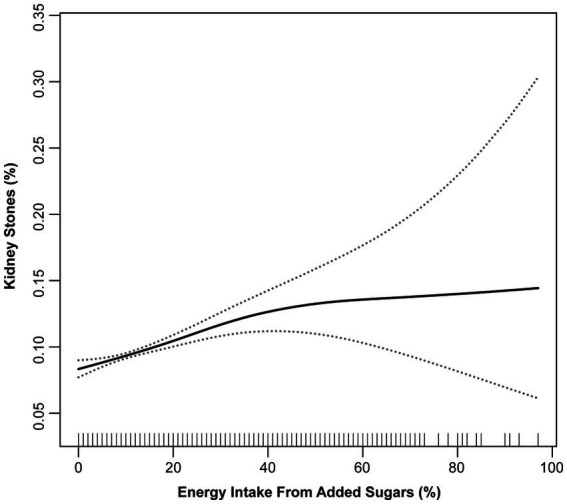
Association of added sugars energy percentage with kidney stone outcome adjusted for all covariates (spline smoothing).

Smooth curve fitting was performed to explore the non-linear association between added sugars energy percentage and kidney stones (Figure 1), which showed a fully-adjusted smooth curve. The piecewise linear regression model and the binary logistic regression model were compared using a likelihood-ratio test. The result of the test showed that there was no significant difference between the two models, as the *p*-value was 0.075 (Table 3).

**Table 3:** Results of binary logistic regression and piecewise linear regression model.^a^

Outcome: kidney stones	Adjusted OR (95% CI)	*p*-value
Fitting by binary logistic regression model	1.01 (1.01, 1.02)	<0.001
*Fitting by piecewise linear regression model*		
Inflection point	30	
<30	1.02 (1.01, 1.02)	<0.001
>30	0.99 (0.97, 1.00)	0.081
Log likelihood ratio test	0.075	

OR, odds ratio; CI, confidence interval; PIR, poverty income ratio; BMI, body mass index; CVD, cardiovascular disease.

a All models were adjusted for gender, age, race, PIR, BMI, education, marital status, smoking, alcohol, energy, HEI-2015, physical activity, gout, diabetes, hypertension, stroke, CVD, cancer, and year cycle.

### Analyses of subgroups and interactions

**Figure 2:**
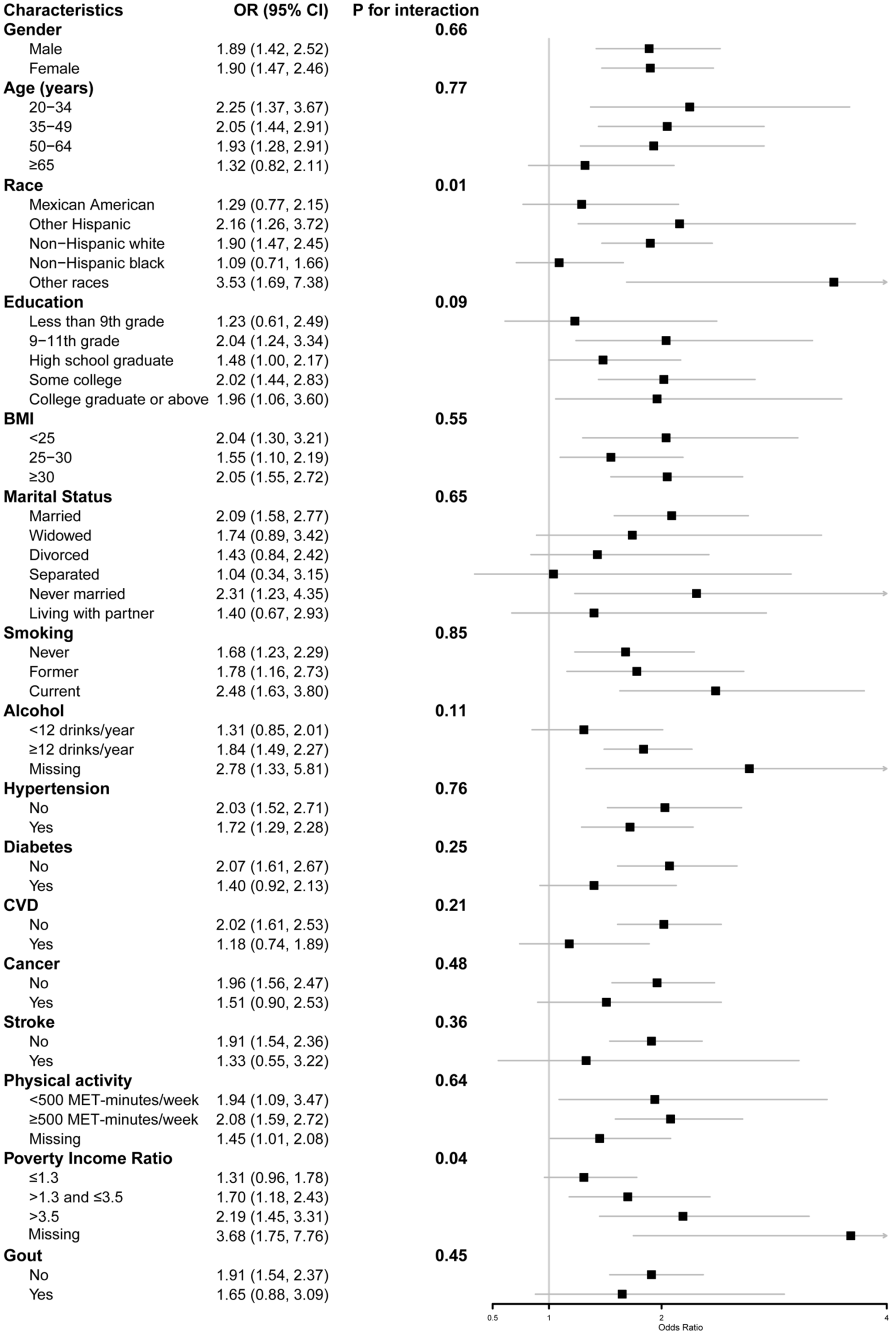
Stratified logistic regression analysis.

Subgroup analyses (Figure 2) found that race and PIR were effect modifiers for the relationship between added sugars and kidney stones after adjustment of other covariates. Results showed the effect sizes of the relationship in different race and PIR levels were significantly different. With regards to race, the odds ratios for the group consuming ≥25% of energy from added sugars, as compared to the group consuming <5% of energy from added sugars, were 1.29 for Mexican American group, 2.16 for other Hispanic group, 1.90 for non-Hispanic white group, 1.09 for non-Hispanic black group, and 3.53 for other races, respectively. The interaction had a *p*-value of 0.01. For the PIR levels, the odds ratios of PIR ≤1.3, >1.3 and ≤3.5, >3.5, and missing group were 1.31, 1.70, 2.19, 3.68, respectively, with p of 0.04 for interaction.

### Sensitivity analysis

This study performed sensitivity multivariate logistic regression analyses using day 2 dietary recall data and the mean dietary intake over the 2 days. Results showed that added sugar energy percentage was positively correlated with kidney stones in the non-adjusted model, the minimally adjusted model, and the fully adjusted model, which was consistent with the above findings. The results of sensitivity analyses were presented in Supplementary Tables S1 and S2.

## Discussion

This cross-sectional study explored the association between added sugars and the prevalence of kidney stones by analyzing 6 continuous cycles of NHANES data sets. Results showed that a higher percentage of energy intake from added sugars was significantly associated with a higher prevalence of kidney stones after taking into account the potential confounders.

Previous studies showed that higher intake of added sugars was associated with unhealthy dietary patterns (10), which in turn might increase risk of unhealthy outcomes such as diabetes and cardiovascular disease (15). A higher overall diet quality, as measured by the healthy eating index, is associated with lower prevalence of kidney stones (4). In our results, higher energy percentage intake from added sugar also corresponded to higher total daily energy intake and lower HEI scores. However, the multivariable logistic regression results remained consistent when we adjusted for HEI as well as total energy, suggesting that the association between added sugar intake and kidney stones may not be explained by overall diet quality.

Various agencies have inconsistent recommendations for the upper limit of added sugar intake. The IOM recommended upper limit for added sugar intake is 25% of total energy (16), they tend to assume that consuming foods or beverage with high amounts of added sugars results in high calories and low micronutrients. Study has also shown that a high intake of added sugars can lead to nutrient dilution in older adults (24). Some micronutrients are closely related to kidney stones, such as calcium and vitamin D (25, 26). However, literature shows that there is insufficient evidence and inconsistence results on the relationship between added sugars and micronutrient intake, and no clear evidence on micronutrient dilution (27, 28). At the same time, the relationship between added sugar intake and micronutrient intake is highly dependent on the food consumed, which may partly explain the inconsistency between the results of the various studies (28). These differences may be closely related to the sources of different sugars. Although most of the added sugars in the United States are derived from SSBs, it is unclear whether the positive correlation between added sugars and kidney stones is caused by SSBs or other food sources. In some literature, food sources of sugars are divided into three categories: treats, toppings, and SSBs (29). In other literature, food sources of sugars are divided into seven categories: dairy products, other milk-based desserts, sugary cereals, cookies/cakes and pastries, sugary products, fruit, and sugary drinks (30). Different food sources correspond to different health outcomes. Fructose intake has found to be independently associated with an increased risk of kidney stones (31). Additionally, a study has found a positive correlation between consumption of sugar-sweetened beverages and kidney stones (21). Therefore, more high-quality prospective studies on the specific sources of added sugars and kidney stones are needed.

In addition, stratified analysis results showed that there were interactions between race or PIR and added sugar on kidney stones. Among them, those of other races and those with missing PIR data had the largest effect size, with an OR value greater than 3. This may be due to the high sensitivity of these groups to added sugars, which may increase their risk of adverse health outcomes. Previous studies have shown that added sugar intake varies among ethnic groups (32). Our study also demonstrates that added sugar is associated differently with the prevalence of kidney stones among participants of different ethnicities. This suggests that interventions aimed at reducing added sugar intake should be specifically designed for different ethnic groups. It also indicates the importance of further exploring the detailed classification of other ethnic groups in future research. The reason for the larger effect size for the population with missing PIR data is unclear, however, the direction of the effect size for all groups for the PIR is consistent and is greater than 1, which is consistent with the results indicating a positive association between added sugars and kidney stones.

### Strengths and limitations

Our study has several strengths. Firstly, this study is based on a nationally representative large population analysis of the association between added sugars and the prevalence of kidney stones, although it is a cross-sectional study rather than a prospective one. Secondly, this research adjusted for a wide range of potential confounding variables, making the results more robust. In addition, we used the percentage of added sugar energy intake rather than the absolute intake of added sugar as the exposure factor, which helps to balance the bias due to individual differences.

The present study has several potential limitations. Firstly, due to the cross-sectional study design, we cannot draw a causal relationship between the percentage of added sugar energy intake and the prevalence of kidney stones because we cannot determine the sequence of sugar intake and kidney stone occurrence. Secondly, while the NHANES data on kidney stones and dietary recall data are self-reported questionnaires, and strict quality control is carried out by professionals, there may still be a certain degree of recall bias, which may impact the results. Furthermore, even after adjusting for some probable confounding factors, we are still unable to completely eliminate the potential confounding effects of some unknown variables. In addition, there is no data on the composition of kidney stones in the NHANES database, such as calcium oxalate stones, uric acid stones, and the relationship between kidney stones of a certain composition and added sugar cannot be evaluated. Moreover, the study was unable to determine the temporal sequence between added sugar intake and the occurrence of kidney stones, and it is also possible that individual added sugar intake occurred after the onset of kidney stones. Finally, this article does not provide a detailed classification of the food or beverage sources of added sugars, nor does it subdivide the types of added sugars, such as monosaccharides, disaccharides or other saccharides, so it is impossible to clarify the relationship between different sources and different classifications of added sugars and kidney stones.

## Conclusion

Consuming a higher percentage of energy from added sugars is positively associated with a higher prevalence of kidney stones. This article provides cross-sectional evidence for the relationship between added sugars and health outcomes. Nevertheless, more high-quality prospective studies are needed to clarify the causal relationship between them.

## Data availability statement

Publicly available datasets were analyzed in this study. This data can be found here: https://www.cdc.gov/nchs/nhanes/index.htm.

## Ethics statement

The studies involving human participants were reviewed and approved by National Center for Health Statistics (NCHS) research ethics review board. The patients/participants provided their written informed consent to participate in this study.

## Author contributions

YB and SY: conceptualization and methodology. SY, ZY, and PZ: data acquisition. SY, ZY, PZ, ZD, XY, and TT: software and formal analysis. SY, ZY, and YB: writing—original draft. SY, ZY, PZ, ZD, XY, TT, and YB: writing—review and editing. SY: data curation and supervision. All authors contributed to the article and approved the submitted version.

## Funding

This work was supported by the Doctoral Fund Project of North Sichuan Medical College (grant number: CBY22-QDA26).

## Conflict of interest

The authors declare that the research was conducted in the absence of any commercial or financial relationships that could be construed as a potential conflict of interest.

## Publisher’s note

All claims expressed in this article are solely those of the authors and do not necessarily represent those of their affiliated organizations, or those of the publisher, the editors and the reviewers. Any product that may be evaluated in this article, or claim that may be made by its manufacturer, is not guaranteed or endorsed by the publisher.

## Footnotes

^1^ https://www.cdc.gov/nchs/nhanes/index.htm
