# Supplementary material for: Association between added sugars and kidney stones in U.S. adults: data from National Health and Nutrition Examination Survey 2007–2018
Source: Front Nutr. 2023 Aug 4;10:1226082. doi: 10.3389/fnut.2023.1226082 (PMC10436224; doi:10.3389/fnut.2023.1226082)
Supplement: Supplementary file 1 [file Data_Sheet_1.DOCX]

**Contents**

[**Figure S1** Flowchart of the study population 2](#_Toc128251706)

[**Table S1** Association of added sugars energy percentage (categorized by 5%, 10%, 25%) with kidney stones (day 2 dietary recall) 2](#_Toc128251707)

[**Table S2** Association of added sugars energy percentage (mean of two days, categorized by 5%, 10%, 25%) with kidney stones 3](#_Toc128251708)

**Figure S1** Flowchart of the study population

**
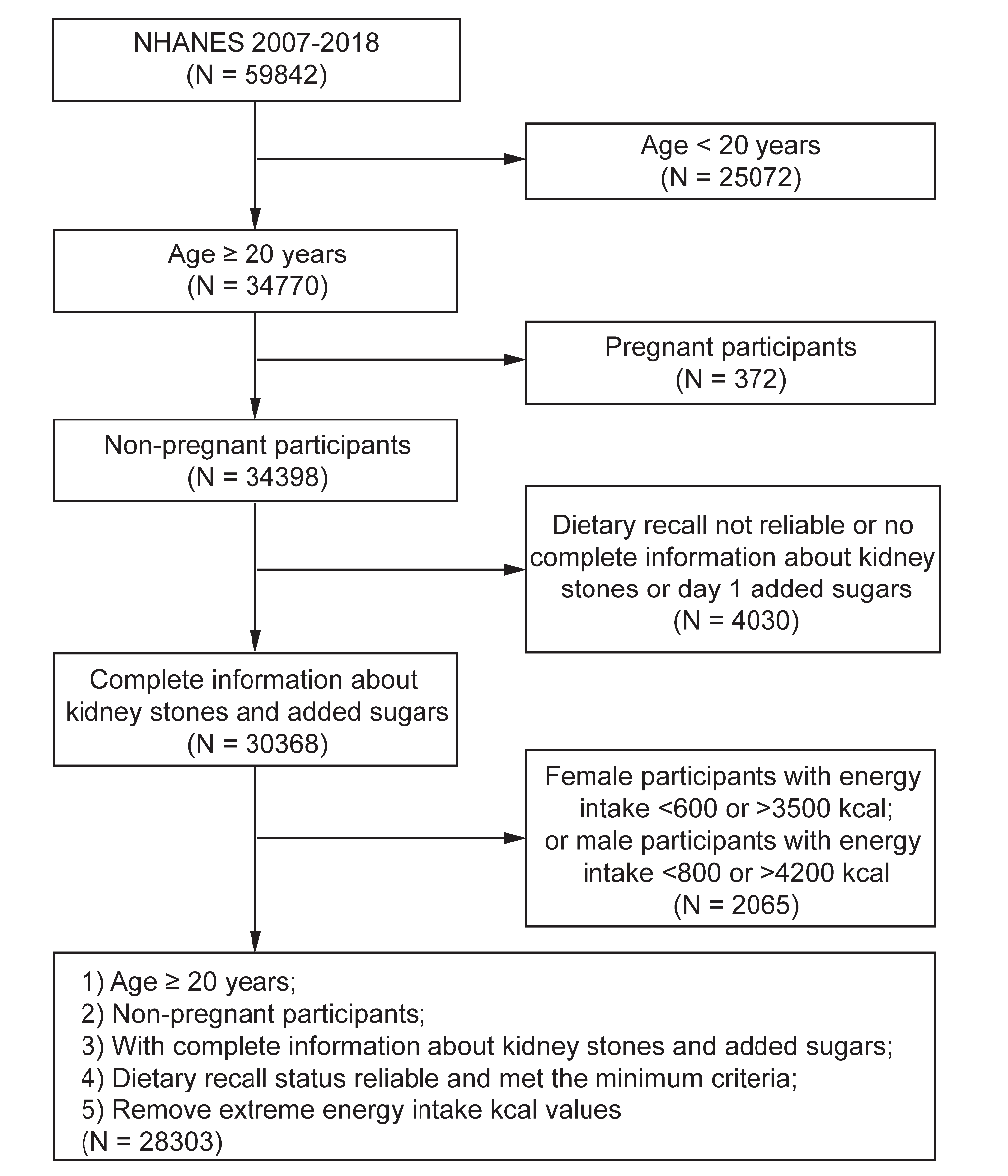
**

**Table S1** Association of added sugars energy percentage (categorized by 5%, 10%, 25%) with kidney stones (day 2 dietary recall)

| Exposure | Model 1^a^ | Model 2^b^ | Model 3^c^ |
| --- | --- | --- | --- |
| %kcal added sugars (continuous) | 1.01 (1.00, 1.01) | 1.01 (1.01, 1.02) | 1.01 (1.01, 1.02) |
| %kcal added sugars (categories) |  |  |  |
| <5 | 1.0 (Ref) | 1.0 (Ref) | 1.0 (Ref) |
| 5-10 | 1.21 (1.03, 1.42) | 1.18 (1.01, 1.39) | 1.26 (1.06, 1.49) |
| 10-25 | 1.21 (1.06, 1.38) | 1.25 (1.09, 1.42) | 1.31 (1.13, 1.52) |
| >25 | 1.39 (1.14, 1.69) | 1.62 (1.37, 1.98) | 1.57 (1.27, 1.94) |
| P value for trend | 0.001 | <0.001 | <0.001 |

^a^ Non-adjusted model: adjusted for None
^b^ Minimally adjusted model: adjusted for gender, age, race

^c^ Fully adjusted model: adjusted for gender, age, race, PIR, BMI, education, marital status, smoking, alcohol, energy (day 2), HEI-2015 (day 2), physical activity, gout, diabetes, hypertension, stroke, CVD, cancer, and year cycle.

Abbreviations: PIR, poverty income ratio; BMI, body mass index; CVD, cardiovascular disease; Ref, reference.

**Table S2** Association of added sugars energy percentage (mean of two days, categorized by 5%, 10%, 25%) with kidney stones

| Exposure | Model 1^a^ | Model 2^b^ | Model 3^c^ |
| --- | --- | --- | --- |
| %kcal added sugars (continuous) | 1.01 (1.01, 1.02) | 1.02 (1.01, 1.03) | 1.02 (1.01, 1.02) |
| %kcal added sugars (categories) |  |  |  |
| <5 | 1.0 (Ref) | 1.0 (Ref) | 1.0 (Ref) |
| 5-10 | 1.03 (0.85, 1.25) | 1.03 (0.84, 1.25) | 1.10 (0.90, 1.34) |
| 10-25 | 1.13 (0.96, 1.31) | 1.22 (1.04, 1.42) | 1.26 (1.06, 1.51) |
| >25 | 1.50 (1.18, 1.90) | 1.82 (1.43, 2.30) | 1.71 (1.34, 2.20) |
| P value for trend | <0.001 | <0.001 | <0.001 |

^a^ Non-adjusted model: adjusted for None
^b^ Minimally adjusted model: adjusted for gender, age, race

^c^ Fully adjusted model: adjusted for gender, age, race, PIR, BMI, education, marital status, smoking, alcohol, energy (mean value of two days), HEI-2015 (mean value of two days), physical activity, gout, diabetes, hypertension, stroke, CVD, cancer, and year cycle.

Abbreviations: PIR, poverty income ratio; BMI, body mass index; CVD, cardiovascular disease; Ref, reference.
